# Efficiency of ectopic thymectomy by three surgical approaches in non-thymomatous myasthenia gravis

**DOI:** 10.1007/s13304-022-01295-5

**Published:** 2022-06-23

**Authors:** Shuishen Zhang, Zhenguang Chen, Bin Li, Chunhua Su, Haoshuai Zhu, Beilong Zhong, Jianyong Zou

**Affiliations:** 1 Department of Thoracic Surgery, The First Affiliated Hospital of Sun Yat-Sen University, Guangzhou, 510080 People’s Republic of China; 2 Department of Cardiothoracic Surgery, Huangpu Branch of the First Affiliated Hospital of Sun Yat-Sen University, Guangzhou, 510700 People’s Republic of China; 3 Clinical Trials Unit, The First Affiliated Hospital of Sun Yat-Sen University, Guangzhou, 510080 People’s Republic of China; 4 Department of Thoracic Surgery, The Fifth Affiliated Hospital of Sun Yat-Sen University, Zhuhai, 519000 People’s Republic of China

**Keywords:** Myasthenia gravis, Surgical approaches, Ectopic thymectomy

## Abstract

**Background:**

To explore the efficiency of ectopic thymectomy by the three surgical approaches of trans-sternum, right unilateral thoracoscopy and thoracoscopic subxiphoid in patients with non-thymomatous myasthenia gravis.

**Methods:**

155 consecutive non-thymomatous myasthenia gravis patients who underwent extended thymectomy by 3 approaches including trans-sternum, right unilateral thoracoscopy and thoracoscopic subxiphoid in 1st affiliated hospital of Sun Yat-Sen University from January 2017 to October 2019 were reviewed. Differences of perioperative clinical characteristics in three surgical approaches were analyzed.

**Results:**

Time to onset of myasthenia gravis (early or late) (*p* = 0.018), blood loss (*p* < 0.001), duration of operation (*p* = 0.031), duration and volume of thoracic drainage (*p* = 0.039 and *p* = 0.026), length of hospitalization (*p* = 0.039), the efficiency of ectopic thymectomy (*p* = 0.037), and the detection rate of ectopic thymus in the second quadrant (*p* = 0.018) were different among the three surgical approaches. In univariate logistic regression analysis, higher efficiency of ectopic thymectomy were associated with transsternal (OR 2.36, 95% CI 1.32–4.22, *p* = 0.011) and thoracoscopic subxiphoid approaches (OR 2.07, 95% CI 1.12–3.82, *p* = 0.033). In the multiple logistic regression analysis, the transsternal approach (OR 2.02, 95% CI 1.10–3.71, *p* = 0.024) was an independent protective factor for the efficiency of ectopic thymectomy.

**Conclusions:**

Both the right unilateral thoracoscopic and thoracoscopic subxiphoid approaches have advantages over the transsternal approach in short-term postoperative recovery. Transsternal approach is still the best choice for ectopic thymectomy while thoracoscopic subxiphoid approach show the potential as an alternative way.

## Introduction

Myasthenia gravis (MG) is an autoimmune disease mediated by antibodies and complement, involving the neuro-muscular junction acetylcholine receptors (AChRs) on the postsynaptic membrane [1]. Extended thymectomy which defined as thymectomy including adipose tissue clearance in anterior mediastinum has been considered as standard surgical treatment for MG [2]. The purpose of removing adipose tissue is to remove the ectopic thymus (ET), as residue of ET is thought to be contributed to poor response to surgical treatment [3].

Transsternal approach has been applied in extended thymectomy for decades [4]. With introduction of video-assisted thoracic surgery technique, thoracoscopic mini-invasive approaches have been chosen as alternative ways for extended thymectomy. However, whether the use of thoracoscopic approach, especially its ability to remove ET, can be as effective as the transsternal one is still controversial. Therefore, we retrospectively analyzed clinical characteristics and the efficiency of ectopic thymectomy in non-thymomatous MG patients who underwent extended thymectomy by three different surgical approaches (trans-sternum, right unilateral thoracoscopy and thoracoscopic subxiphoid).

## Patients and methods

The study protocol was approved by the Ethics Review Board of the First Affiliated Hospital of Sun Yat-Sen University and conformed to the Declaration of Helsinki.

155 MG patients who underwent extended thymectomy at the First Affiliated Hospital of Sun Yat-Sen University, Guangzhou, China, from January 2017 to October 2019 were reviewed. All patients underwent extended thymectomy in approaches of trans-sternum (TS), right unilateral thoracoscopy (RUT) or thoracoscopic subxiphoid (TSX). The surgeries were performed by three sophisticated surgeons. Doctor Zou performed TS, RUT and TSX in 18, 16, and 23 patients. Doctor Chen performed the three approaches in 22, 13 and 16 patients while Doctor Su performed those in 12, 21 and 14 cases, respectively. The differences of approaches chosen among three doctors was not significant (not shown in the article). The basic surgical procedure included complete resection of the thymus and clearance of adipose tissue in the anterior mediastinum. The range of adipose tissue clearance included bilateral to the phrenic nerve, up to the root of neck, and down to both sides of the cardio-diaphragmatic angle [5].

Clinical characteristics of age at surgery, sex, Myasthenia Gravis Foundation of America (MGFA) stage, pyridostigmine dose, time from disease onset to surgery, preoperative and postoperative myasthenic crisis, AchRs level, preoperative and postoperative immunoglobulin treatment, preoperative and postoperative plasmapheresis, perioperative applications of corticosteroid, surgical approaches, blood loss, operation time, length and volume of thoracic drainage, ratio of thymic parenchyma, incidence of ET removal, distribution of ET, time of hospitalization and postoperative complications were collected.

**Fig. 1:**
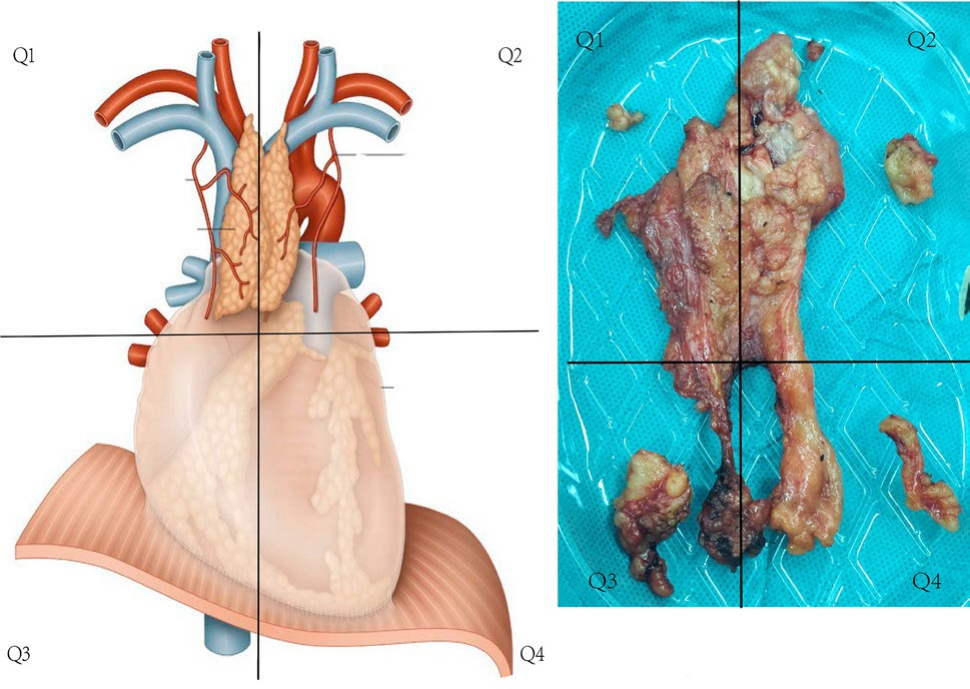
Four quadrants of anterior mediastinum and root of the neck which were separated by the anterior midline and the horizontal line at the start of the ascending aorta. Q1: Upper mediastinum and root of neck on the right; Q2: Upper mediastinum and root of neck on the left; Q3: Lower mediastinum and cardiophrenic angle on the right; Q4: Lower mediastinum and cardiophrenic angle on the left

In this study, we simplified the ET distribution area into four quadrants using the anterior midline and the horizontal line at the start of the ascending aorta (Fig. 1). Adipose tissues removed in the quadrants were placed in buffered 10% formalin and labelled. They were then sliced and stained using haematoxilin and eosin. Final specimens were independently examined by two pathologists. ET was diagnosed as the un-encapsulated lobules of thymus, microscopic foci of thymic tissue or the Hassals corpuscles in adipose tissues [6]. Ratio of thymic parenchyma, which defined as volume of thymic parenchyma comparing to that of adipose tissue in the thymus, was also assessed by the pathologists. According to the ratio of thymic parenchyma, we categorized the patients into three groups (“< 40%”, “≥ 40% and “< 70%” and “≥ 70%”).

Preoperative and postoperative myasthenic crisis which were defined as the complication characterized by worsening of muscle weakness resulting in respiratory failure presented within 1 month before surgery and 2 weeks after surgery. The application of Immunoglobin and plasmapheresis within 1 week before and 2 weeks after surgery was considered as perioperative treatment.

The median follow-up time was 27 months. According to MG symptoms, the patients were categorized into complete remission (CR), partial remission (PR), unchanging (UC) and progression disease (PD). The assessment should be performed and agreed by both the surgeon and neurologist.

### Statistical analysis

Quantitative data were reported as mean ± standard deviation (SD) and compared by one-way ANOVA test. Qualitative data were reported as frequency (ratio) and compared by chi-square test or Fisher’s exact test. Univariate and multivariate logistic regression analysis (binomial) were applied to identify association between clinical factors and the efficiency of ectopic thymectomy. A *p* value ≤ 0.05 was considered significant. All statistical analyses were performed using IBM SPSS Statistics, version 22 (IBM Corp, Armonk, NY, USA).

## Results

The study included a total of 155 patients, with 60.7% female and 39.3% male. Mean age at surgery was 27.3 ± 9.29 years old, and juvenile patients (< 18 years old) were accounted for 12.9%.

Depending on MGFA stage, 1 case (0.7%), 43 cases (27.7%), 40 cases (25.8%), 38 cases (24.5%), 27 cases (17.4%), 4 cases (2.6%) and 2 cases (1.2%) were at stage I, IIA, IIB, IIIA, IIIB, IVA and IVB, respectively. All patients were positive for AchRs antibodies. 11 patients (7.1%) experienced postoperative myasthenic crisis. The mean time from disease onset to surgery was 18.1 ± 6.20 months. All patients were treated with pyridostigmine (225.68 ± 53.22 mg/day); 113 (72.9%) cases were perioperatively treated with glucocorticoid. Before surgery, 31 (20.0%) and 13 (8.4%) were treated with immunoglobulin and plasmapheresis respectively.

Surgical approaches of TS, RUT and TSX were performed in 57 (36.8%), 51 (32.9%) and 47 (30.3%) patients, respectively. Mean volume of blood loss was 103.3 ± 63.04 ml and mean time of operation was 146.3 ± 62.41 min. Mean length and volume of thoracic drainage were 2.63 ± 1.22 days and 269.93 ± 146.32 ml, respectively.

ET were found in 64 (41.3%) cases. ET appeared in Q1, Q2, Q3 and Q4 quadrants were 26 (40.0%), 36 (55.4%), 22 (33.4%) and 29 (44.6%), respectively. Ratio of thymic parenchyma of “40%”, “ > = 40% and < 70%” and “ > = 70%” were found in 72 (46.5%), 71 (45.8%) and 12 (7.7%) patients.

Postoperatively, 29 (18.7%) cases were treated with immunoglobin and 6 (3.9%) cases underwent plasmapheresis. 22 cases (8.2%) experienced postoperative myasthenic crisis. Mean time of hospitalization was 9.61 ± 3.91 days. 31 (20%) patients had postoperative complications, among them 13 (41.9%) were surgical incision infection, 14 (45.2%) were pneumonia and 9 (29.0%) were mediastinitis.

The follow-up data showed the patients with CR, PR, UC and PD were 9 (5.8%), 100 (64.5%), 36 (23.2%) and 10 (6.5%), respectively (Table 1).

**Table 1:** Clinical and pathological characteristics of MG patients underwent extended thymectomy

Characteristic	*N*	%
Number of patients	155	100%
Age, years, mean ± SD	27.33 ± 9.29	
Juvenile (< 18 years)	20	12.9%
Adults (>= 18 years)	135	87.1%
MG onset		
Early (< 40 years)	138	89.0%
Late (>= 40 years)	17	11.0%
Sex		
Male	61	39.4%
Female	94	60.7%
MGFA stage		
I	1	0.7%
IIA	43	27.7%
IIB	40	25.8%
IIIA	38	24.5%
IIIB	27	17.4%
IVA	4	2.6%
IVB	2	1.3%
AchR level		
Positive	155	100.0%
Negative	0	0.0%
Pulmonary function		
FEV1 (mean ± SD)	66.5% ± 8.8%	
FVC (mean ± SD)	74.2% ± 10.3%	
Preoperative myasthenic crisis		
Positive	11	7.1%
Negative	144	92.9%
Time between disease onset and surgery (months)		
Mean ± SD	18.81 ± 6.20	
Pyridostigmine dose (mg)		
Mean ± SD	225.68 ± 53.22	
Perioperative glucocorticoid usage		
Positive	113	72.9%
Negative	42	27.1%
Preoperative immunoglobin usage		
Positive	31	20.0%
Negative	124	80.0%
Preoperative plasmapheresis		
Positive	13	8.4%
Negative	142	91.6%
Surgical approach		
TS	57	36.8%
RUT	51	32.9%
TSX	47	30.3%
Blood loss		
Mean ± SD	103.30 ± 63.04	
< 50 ml	33	21.3%
50–100 ml	41	26.5%
> 100 ml	81	52.2%
Operation duration (min)		
Mean ± SD	146.30 ± 62.41	
< 60 min	5	3.2%
60–120 min	71	45.8%
> 120 min	79	51.0%
Length of chest tube drainage (days)		
Mean ± SD	2.63 ± 1.22	
Volume of chest tube drainage (ml)		
Mean ± SD	269.93 ± 146.32	
ETT		
Positive	64	41.3%
Negative	91	58.7%
Distribution of ETT		
Q1	26	40.0%
Q2	36	55.4%
Q3	22	33.9%
Q4	29	44.6%
Ratio of thymus parenchyma		
< 40%	72	46.5%
>= 40% and < 70%	71	45.8%
>= 70%	12	7.7%
Postoperative immunoglobin usage		
Positive	29	18.7%
Negative	126	81.3%
Postoperative plasmapheresis		
Positive	6	3.9%
Negative	149	96.1%
Postoperative myasthenic crisis		
Positive	22	8.2%
Negative	133	91.8%
Length of hospital stay (days)		
Mean ± SD	9.61 ± 3.91	
Postoperative complications		
Surgical incision infection	13	41.9%
Pneumonia	14	45.2%
Mediastinitis	9	29.0%
None	124	80.0%
Prognosis		
CR	9	5.8%
PR	100	64.5%
UC	36	23.2%
PD	10	6.5%

*MG* myasthenia gravis, *MGFA* Myasthenia Gravis Foundation of America, *TS* transsternal, *RUT* right unilateral thoracoscopy, *TSX* thoracoscopic subxiphoid, *ETT* ectopic thymic tissue, *CR* complete remission, *PR* partial remission, *UC* unchanging, *PD* progression disease

Comparison of clinical characteristics in the three surgical approaches groups (TS vs. RUT vs. TSX) showed volume of blood loss (145.3 ml vs. 87.1 ml vs. 70 ml, *p* <  **0.001**), operation time (159.5 min vs. 128.4 min vs. 149.8 min, *p* = **0.031**), length of thoracic drainage (2.9 days vs. 2.5 days vs. 2.4 days, *p*** = 0.047**), volume of thoracic drainage (306.7 ml vs. 266.3 ml vs. 229.3 ml, *p* =  **0.026**), incidence of ET removal (10 (43.5%) vs. 13 (25.5%) vs. 20 (42.5%), *p* = 0.037), probability of ET found in Q2 quadrant (18 (64.3%) vs. 3 (23.1%) vs. 13 (65.0%), *p* =  **0.032**) and time of hospitalization (10.51 days vs. 9.57 days vs. 8.55 days, *p* =  **0.039**) were significantly different.

In TS group, the patients with CR, PR, UC and PD were 4 (7%), 38 (66.7%), 12 (21%) and 3 (5.3%). In RUT group, the patients with CR, PR, UC and PD were 2 (3.9%), 32 (62.6%), 14 (27.5%) and 3 (6%). In TSX group, the patients with CR, PR, UC and PD were 3 (6.4%), 30 (63.8%), 10 (21.3%) and 4 (8.5%), respectively. The differences of prognosis among three surgical approaches were not obvious statistically (Table 2).

**Table 2:** Comparison of clinical characteristics in three surgical approaches

Variables	TS	RUT	TSX	*p* values
Age				
Mean ± SD	28.40 ± 10.24	28.35 ± 9.98	24.91 ± 6.67	0.102
Juvenile (< 18)	5 (8.8%)	9 (17.7%)	6 (12.8%)	0.389
Adults (>= 18)	52 (91.2%)	42 (82.3%)	41 (87.2%)	
Sex				
Male	25 (43.9%)	22 (43.1%)	14 (30.0%)	0.273
Female	32 (56.1%)	29 (56.9%)	35 (70.0%)	
MG onset				
Early (< 40 years)	51 (89.5%)	41 (80.4%)	46 (97.9%)	**0.018**
Late (>= 40 years)	6 (10.5%)	10 (19.6%)	1 (2.13%)	
MGFA stage				
I	0 (0%)	0 (0%)	1 (2.13%)	0.408
IIA	17 (29.5%)	13 (25.5%)	13 (27.7%)	
IIB	9 (15.8%)	19 (37.3%)	12 (25.5%)	
IIIA	17 (29.5%)	10 (19.6%)	11 (23.4%)	
IIIB	11 (19.3%)	7 (13.7%)	9 (19.2%)	
IVA	1 (1.7%)	2 (3.9%)	1 (2.13%)	
IVB	2 (3.5%)	0 (0%)	0 (0%)	
Pyridostigmine dose (mg)				
Mean ± SD	217.89 ± 55.99	224.12 ± 52.08	236.81 ± 50.13	0.191
Time between disease onset and surgery (months)				
Mean ± SD	18.96 ± 5.68	19.22 ± 5.08	18.17 ± 7.80	0.814
Pulmonary function (%)				
FEV1 (mean ± SD)	67.66 ± 7.82	65.50 ± 11.06	66.32 ± 6.97	0.439
FVC (mean ± SD)	76.02 ± 9.13	72.43 ± 12.59	73.77 ± 8.58	0.185
Preoperative myasthenic crisis				
Positive	4 (7%)	5 (9.8%)	2 (4.3%)	0.628
Negative	53 (93%)	46 90.2%)	45 (95.7%)	
Preoperative immunoglobin treatment				
Positive	16 (28.1%)	9 (17.7%)	6 (12.8%)	0.133
Negative	41 (71.9%)	42 (82.3%)	41 (87.2%)	
Preoperative plasmapheresis				
Positive	6 (10.5%)	5 (9.8%)	2 (4.3%)	0.512
Negative	51 (89.5%)	46 (90.2%)	45 (95.7%)	
Perioperative glucocorticoid usage				
Positive	40 (70.2%)	37 (72.5%)	36 (76.6%)	0.762
Negative	17 (29.8%)	14 (27.5%)	11 (23.4%)	
Blood loss (ml)				
Mean ± SD	145.79 ± 57.57	86.47 ± 55.67	70 ± 47.09	** < 0.001**
< 50 ml	1 (1.7%)	13 (25.5%)	19 (40.4%)	** < 0.001**
50–100 ml	11 (19.3%)	17 (33.3%)	13 (27.7%)	
> 100 ml	15 (79%)	21 (41.2%)	15 (31.9%)	
Operation duration (min)				
Mean ± SD	159.5 ± 61.05	128.43 ± 56.79	149.78 ± 66.55	**0.031**
< 60 min	0 (0%)	2 (3.9%)	3 (6.4%)	0.146
60–120 min	24 (42.1%)	28 (54.9%)	19 (40.4%)	
> 120 min	33 (57.9%)	21 (41.2%)	25 (53.2%)	
Length of chest tube drainage (days)				
Mean ± SD	2.95 ± 1.38	2.49 ± 1.05	2.38 ± 1.13	**0.039**
Volume of chest tube drainage (ml)				
Mean ± SD	311.75 ± 140.71	260.59 ± 135.93	229.36 ± 153.43	**0.026**
Ratio of thymus parenchyma				
< 40%	33 (57.9%)	18 (35.3%)	21 (44.7%)	0.149
>= 40% and < 70%	22 (38.6%)	27 (52.9%)	22 (46.8%)	
>= 70%	2 (3.5%)	6 (11.76%)	4 (8.5%)	
ETT				
Positive	28 (49.1%)	11 (25.5%)	22 (46.8%)	**0.005**
Negative	29 (50.9%)	38 (74.5%)	26 (53.2%)	
Distribution of ETT				
Q1	12 (41.4%)	8 (57.1%)	6 (27.3%)	0.200
Q2	19 (65.5%)	3 (21.4%)	13 (63.6%)	**0.018**
Q3	9 (31.0%)	7 (50.0%)	5 (27.27%)	0.340
Q4	12 (41.4%)	6 (42.9%)	11 (50.0%)	0.819
Postoperative immunoglobin usage				
Positive	13 (22.8%)	9 (17.7%)	7 (14.9%)	0.572
Negative	44 (77.2%)	42 (82.3%)	40 (85.1%)	
Postoperative plasmapheresis				
Positive	2 (3.5%)	2 (3.9%)	2 (4.3%)	1.00
Negative	55 (96.5%)	49 (96.1%)	45 (95.7%)	
Postoperative myasthenic crisis				
Positive	6 (10.5%)	9 (17.7%)	7 (14.9%)	0.563
Negative	51 (89.5%)	42 (82.3%)	40 (85.1%)	
Length of hospital stay (days)				
Mean ± SD	10.61 ± 3.93	9.45 ± 4.12	8.55 ± 3.41	**0.039**
Postoperative complications				
Surgical incision infection	7 (12.3%)	1 (2.0%)	5 (10.6%)	0.105
Pneumonia	5 (8.8%)	6 (11.76%)	3 (6.4%)	0.687
Mediastinitis	6 (10.5%)	1 (2.0%)	2 (4.3%)	0.154
None	42 (73.58%)	44 (86.3%)	38 (80.9%)	0.26
Prognosis				
CR	4 (7%)	2 (3.9%)	3 (6.4%)	0.667
PR	38 (66.7%)	32 (62.6%)	30 (63.8%)	
UC	12 (21%)	14 (27.5%)	10 (21.3%)	
PD	3 (5.3%)	3 (6%)	4 (8.5%)	

*MG* myasthenia gravis, *MGFA* Myasthenia Gravis Foundation of America, *TS* transsternal, *RUT* right unilateral thoracoscopy, *TSX* thoracoscopic subxiphoid, *ETT* ectopic thymic tissue, *CR* complete remission, *PR* partial remission, *UC* unchanging, *PD* progression disease

Univariate logistics regression test (binomial) revealed surgical approaches of TS (OR 2.36, 95% CI 1.32–4.22, *p* =  **0.011**) and TSX (OR 2.07, 95% CI 1.12–3.82, *p* =  **0.033**) both had higher efficiency of ectopic thymectomy (Table 3). While multivariate logistics regression test (binomial) revealed only TS had higher efficiency of ectopic thymectomy (OR 2.02, 95% CI 1.10–3.71, *p* = **0.024**) (Table 4).

**Table 3:** Univariate Logistic Regression Analysis (Binomial) of Risk Factors associated with ETT Removal in Non-thymomatous MG Patients

Variables	OR	95% confidence interval	P-value
Sex			
Male	1.00		
Female	1.33	0.88–2.00	0.175
Age at surgery (years)	0.99	0.97–1.02	0.603
Juvenile or adult			
Juvenile	1.00		
Adult	1.21	0.64–2.26	0.559
MG onset			
Early	1.00		
Late	0.69	0.32–1.47	0.336
MGFA stage			
Stage IA + IIA	1.00		
Stage IIB	0.72	0.41–1.24	0.233
Stage IIIA	1.04	0.65–1.66	0.862
Stage IIIB + IVA + IVB	0.87	0.51–1.48	0.599
Preoperative Myasthenic Crisis			
Yes	1.00		
No	1.55	0.58–4.15	0.380
Time between disease onset and surgery (months)	1.01	0.98–1.04	0.436
FEV1 (%)	1.00	0.98–1.01	0.751
FVC (%)	1.00	0.98–1.02	0.767
Pyridostigmine dose (mg)	1.00	0.99–1.00	0.967
Perioperative glucocorticoid usage			
No	1.00		
Yes	1.03	0.67–1.57	0.901
Preoperative immunoglobin usage			
Yes	1.00		
No	1.08	0.66–1.77	0.748
Preoperative plasmapheresis			
Yes	1.00		
No	0.88	0.48–1.64	0.699
Surgical approach			
TrUL	1.00		
TS	2.36	1.32–4.22	**0.011**
TSX	2.07	1.12–3.82	**0.033**
Blood loss (ml)	1.00	0.99–1.00	0.959
Operation duration (min)	1.00	0.99–1.00	0.402
Ratio of thymus parenchyma			
< 40%	1.00		
> = 40% and < 70%	0.83	0.59–1.22	0.347
> = 70%	0.73	0.31–1.68	0.457

*ETT* Ectopic thymic tissue,* MG* Myasthenia Gravis,* MGFA* Myasthenia Gravis Foundation of America,* TS* Transsternal,* TrUL* Thoracoscopic Right Unilateral,* TSX* Thoracoscopic Subxiphoid

**Table 4:** Multivariate Logistic Regression Analysis (binomial) of Risk Factors associated with ETT Removal in Non-thymomatous MG Patients

Variables	OR	95% confidence interval	P-value
Sex			
Male	1.00		
Female	1.27	0.81–1.89	0.327
Age at surgery (years)	0.99	0.96–1.02	0.622
Juvenile or adult			
Juvenile	1.00		
Adult	1.16	0.54–2.53	0.700
MG onset			
Early	1.00		
Late	0.99	0.40–2.46	0.979
MGFA stage			
Stage IA + IIA	1.00		
Stage IIB	0.86	0.49–1.50	0.588
Stage IIIA	1.04	0.66–1.64	0.867
Stage IIIB + IVA + IVB	0.88	0.51–1.51	0.643
Preoperative Myasthenic Crisis			
Yes	1.00		
No	1.2	0.40–3.42	0.731
Time between disease onset and surgery (months)	1.01	0.98–1.04	0.390
Perioperative glucocorticoid usage			
No	1.00		
Yes	0.96	0.63–1.48	0.861
Preoperative immunoglobin usage			
Yes	1.00		
No	1.11	0.69–1.80	0.660
Preoperative plasmapheresis			
Yes	1.00		
No	0.79	0.43–1.59	0.574
Surgical approach			
TrUL	1.00		
TS	2.02	1.10–3.71	**0.024**
TSX	1.67	0.95–2.93	0.076
Blood loss (ml)	1.00	0.99–1.00	0.405
Operation duration (min)	1.00	0.99–1.00	0.629
Ratio of thymus parenchyma			
< 40%	1.00		
> = 40% and < 70%	0.90	0.59–1.38	0.634
> = 70%	0.90	0.40–2.03	0.808

*ETT* Ectopic thymic tissue,* MG* Myasthenia Gravis,* MGFA* Myasthenia Gravis Foundation of America,* TS* Transsternal,* TrUL* Thoracoscopic Right Unilateral,* TSX* Thoracoscopic Subxiphoid

## Discussion

Extended thymectomy is an effective treatment for MG [7]. Complete removal of thymus and adipose tissue in both anterior mediastinum and root of neck is critical of surgery [8]. Ectopic thymectomy is the purpose of adipose tissue clearance, as residual of ET is associated with poor response to extended thymectomy.

Due to excellent exposure in anterior mediastinum, TS has been widely accepted by thoracic surgeons and become the preferred surgical technique for MG treatment. However, patients undergoing thymectomy by TS have to suffer severe surgical wounds and pain. Therefore, it is of great value to find a suitable surgical technique that is as effective as TS but less invasive.

Since the introduction of thoracoscopic mini-invasive surgical technique, many studies have focused on thoracoscopic approaches applied in extended thymectomy [9–11]. Calvin S H Ng considered RUT as the better option because on one hand it had good vision on the junction of superior vena cava and the brachiocephalic vein, on the other hand, it was easier for right-handed surgeons performing thoracoscopic surgery to start at the inferior poles and work cephalad from the right side [12]. But Mineo advocated a left-sided approach and believed from the left the dissection maneuvers are safer, because the superior vena cava lies out of the surgical field, thus reducing risk of accidental injury [13]. In our opinion, Calvin S H Ng’s views is more acceptable, because on one hand, the good exposure conduces to avoid injury, on the other hand ET adjacent to the superior vena cava should not be ignored. However, when we applied RUT, for the better view of contralateral site, we usually needed to compress the heart which might cause arrhythmia or hypotension, even so the views of Q2 and Q4 quadrants were still poor. Chang Young Lee reported that bilateral thoracoscopic thymectomy has a surgical extent similar to that of transsternal approach with more favorable early surgical outcomes [14].

**Fig. 2:**
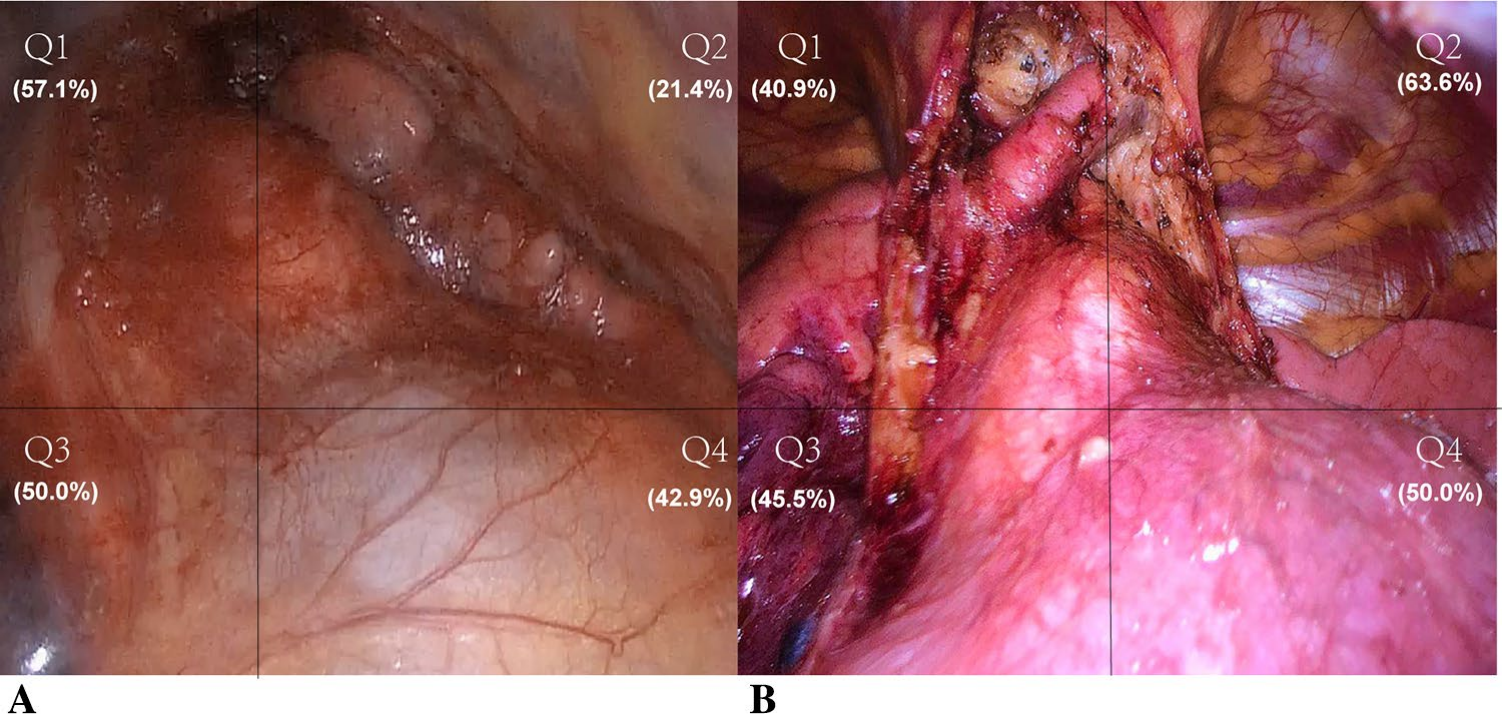
**A** Incisions and view of surgery in right unilateral thoracoscopic approach. View of Q2 and Q4 quadrants was poor and the efficiency of ectopic thymectomy was 25.5%; **B** Incisions and view of surgery in thoracoscopic subxiphoid approach: View of Q2 and Q4 quadrants was better and the efficiency of ectopic thymectomy was 46.8%

Extended thymectomy by TSX was first reported by C-P Hsu in 2002 [15]. He later reported a comparative study showed both RUT and TSX had satisfactory results for MG patients. Though C-P Hsu suggested TSX had better view of the bilateral pleural cavities, he did not mentioned the difference in ectopic thymectomy between RUT and TSX [16]. In this study, techniques of TSX (Three ports) was similar as the one described by Qiang Lu and colleagues in 2018 [17]. Though TSX spent longer operation time comparing with RUT, it had not only both the minimal volume of blood loss and thoracic drainage, but also the shortest time of thoracic drainage and hospitalization among three approaches. More importantly, surgical exposure of TSX was close to TS, especially the excellent view in both Q2 and Q4 quadrants, which was conducive to ectopic thymectomy comparing with RUT (Fig. 2).

ET in MG patients was first reported by Akira Masaoka [6]. Jaretzki III, a further described distribution of ET in 12 regions of anterior mediastinum [18]. Adverse impact of ET on effect of surgery for MG patients have been reported by many studies. Two phenomena support involvement of ET in the pathogenesis of MG. First, germinal centers which were considered being source of MG autoantibodies were also found in ET [19]. Second, several studies found that the presence of ET was significantly associated with poor response of thymectomy for MG [20–22]. Although some other studies have shown that there is no relationship between the discovery of ET and the effect of thymectomy in MG patients [13, 23], Zielinski and colleagues attributed this phenomenon to the residual of ET rather than the presence of it affecting the effect of thymectomy [24].

In this study, ET was found in 64 patients (41.3%), and the odds of finding ET were different in the 3 surgical methods. TS and TSX had close efficiency of ectopic thymectomy (49.1% and 46.8%, *p* = 0.397, not shown in the tables) which was superior to that of RUT (25.5%, *p* = 0.005).

**Fig. 3:**
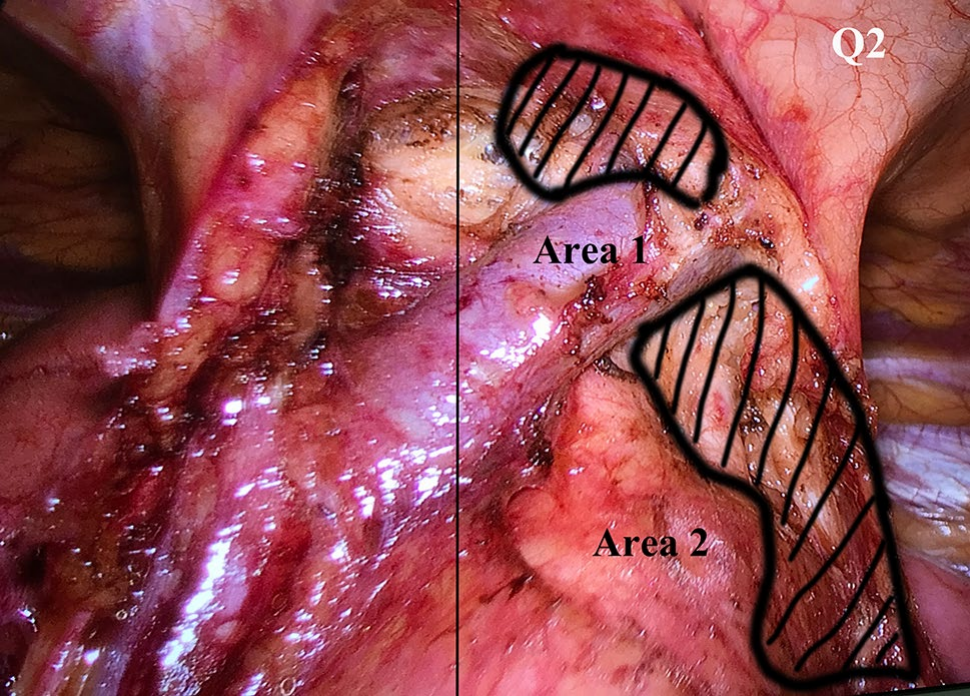
Areas of Q2 quadrant where ectopic thymus commonly distributed in the view of thoracoscopic subxiphoid approach: area 1 was the site above left innominate vein and area 2 was the site between aortic arch and left phrenic nerve

Interestingly, the efficiency of ectopic thymectomy among the three surgical approaches were significantly different only in Q2 quadrant which located in upper left division of anterior mediastinum. In this area, ET were commonly distributed in the space above and below the left innominate vein where was difficult to be revealed by RUT (Fig. 3). As we mentioned above, major defect of RUT was the poor exposure of both Q2 and Q4 quadrants. When thoracic surgeons performed extended thymectomy by RUT, poor vision on the contralateral site might hesitate them from adipose tissue clearance and cause lower efficiency of ectopic thymectomy.

In this study, both TSX and RUT showed advantages of less blood loss and shorter operation time comparing with TS, however, TS had the best efficiency of ectopic thymectomy among the three approaches. Surgical injury caused by TS increased volume and time of thoracic drainage and hospitalization, therefore, it affected short-term recovery of the patients. However, TS also had the best efficiency of ectopic thymectomy which might be in favor of long-term outcome. In our opinion, at present TS is still an indispensable method of extended thymectomy in MG patients and cannot be replaced by thoracoscopic methods, especially unilateral thoracoscopic approach. However, it is noteworthy that TSX and bilateral thoracoscopic approaches have the similar surgical extent as TS. Both of them are potential to be as efficiency as TS in ectopic thymectomy. In this study, the different of ectopic thymectomy between TS and TSX was minor also supported this potentiality. Meanwhile, relative to the bilateral thoracoscopic approach, TSX showed advantage in more simplified procedure and easier conversion to TS while unpredicted injury happened. But nowadays, we still need more evidences to support that TSX could be an alternative way of surgical treatment in MG patients.

Prognosis data showed that overall response (CR + PR) to surgery in all patients was 70.3%. The differences of response rate among three groups were not statistically significant. However, TS and TSX approaches still showed the trend of better outcome comparing to RUT. As the median follow-up time was only less than 3 years, whether surgical approaches and their efficiency of ectopic thymectomy has affection on long-term outcome of MG is still unsettled.

Limitations of this study were shown below. First, it was a retrospective study, suggesting that the reliability of the results may be impaired by clinical characteristics bias. Second, Surgical process and outcome were strongly associated with surgeons’ experience and preference. The bias caused by that should not be ignored. Third, the size of cases was small and the time of follow-up was short, therefore, further study with larger sample with longer follow-up time may help us better understand the influence of surgical approaches on ectopic thymectomy.

Conclusively, Both RUT and TSX showed advantages in short-term recovery over TS in extended thymectomy. For the efficiency of ectopic thymectomy, TS was still the best option while TSX showed potential to be an alternative way and less invasive.
